# Decreasing use of pancreatic necrosectomy and NSQIP predictors of complications and mortality

**DOI:** 10.1186/s13017-022-00462-8

**Published:** 2022-12-12

**Authors:** Amy E. Liepert, George Ventro, Jessica L. Weaver, Allison E. Berndtson, Laura N. Godat, Laura M. Adams, Jarrett Santorelli, Todd W. Costantini, Jay J. Doucet

**Affiliations:** Division of Trauma, Surgical Critical Care, Burns and Acute Care Surgery, Department of Surgery, University of California, San Diego, USA

**Keywords:** Pancreatitis, Pancreatic necrosectomy, Infected pancreatic necrosis, Video-assisted retroperitoneal debridement, Pancreatic pseudocyst

## Abstract

**Background:**

Surgical pancreatic necrosectomy (SPN) is an option for the management of infected pancreatic necrosis. The literature indicates that an escalating, combined endoscopic, interventional radiology and minimally invasive surgery “step-up” approach, such as video-assisted retroperitoneal debridement, may reduce the number of required SPNs and ICU complications, such as multiple organ failure. We hypothesized that complications for surgically treated severe necrotizing pancreatitis patients decreased during the period of adoption of the “step-up” approach.

**Methods:**

The American college of surgeons national surgery quality improvement program database (ACS-NSQIP) was used to find SPN cases from 2007 to 2019 in ACS-NSQIP submitting hospitals. Mortality and Clavien-Dindo class 4 (CD4) ICU complications were collected. Predictors of outcomes were identified by univariate and multivariate analyses.

**Results:**

There were 2457 SPN cases. SPN cases decreased from 0.09% in 2007 to 0.01% in 2019 of NSQIP operative cases (*p* < 0.001). Overall mortality was 8.5% and did not decrease with time. CD4 complications decreased from 40 to 27% (*p* < 0.001). There was a 65% reduction in SPN cases requiring a return to the operating room. Multivariate predictors of complications were emergency general surgery (EGS, *p* < 0.001), serum albumin (*p* < 0.0001) and modified frailty index (mFI) (*p* < 0.0001). Multivariate predictors of mortality were EGS (*p* < 0.0001), serum albumin (*p* < 0.0001), and mFI (*p* < 0.04). The mFI decreased after 2010 (*p* < 0.001).

**Conclusion:**

SPNs decreased after 2010, with decreasing CD4 complications, decreasing reoperation rates and stable mortality rates, likely indicating broad adoption of a “step-up” approach. Larger, prospective studies to compare indications and outcomes for “step up” versus open SPN are warranted.

**Supplementary Information:**

The online version contains supplementary material available at 10.1186/s13017-022-00462-8.

## Background

Severe acute pancreatitis resulted in nearly 250,000 annual US hospital admissions in 2017 and cost an estimated $3.3 billion [1]. Up to 20% of these patients progress to necrotizing pancreatitis or walled-off pancreatic necrosis (WOPN) requiring invasive intervention [2]. Infected pancreatic necrosis has an associated mortality as high as 30–40% [3–5].

Management techniques with surgical pancreatic necrosectomy (SPN) have evolved from early open necrosectomy towards delayed, minimally invasive approaches such as non-surgeon-performed endoscopic transgastric debridement, radiologist-performed percutaneous drainage, and surgeon-performed minimally invasive surgical approaches, such as video-assisted retroperitoneal debridement (VARD) [6]. (Additional file 1: Fig. S1–S3) A delayed, less invasive approach to pancreatic necrosectomy has been associated with reductions in surgical mortality and morbidity [7–9]. A “step-up” approach was promoted in 2010 by the Dutch Pancreatic Study Group’s PANTER trial “(PAncreatitis, Necrosectomy versus sTEp up appRoach),” which compared open necrosectomy to a progressive, minimally invasive surgical approach (MIS) initially using percutaneous drainage and endoscopic debridement. Cases that failed to resolve with this intervention then went on to require MIS procedures such as VARD. PANTER showed decreased morbidity, including lower rates of sepsis and multiple organ failure (MOF), but no significant difference in mortality [10, 11]. Currently, there is no consensus algorithm and significant local practice variation may exist for the management of infected pancreatic necrosis. This may be due to the inherent variety of patient clinical status, anatomy, and contents of WOPN, as well as institutional and surgeon preferences [3, 12].

As the “step-up” approach has become more common, mortality associated with necrotizing pancreatitis has decreased. A 1998–2010 Nationwide Inpatient Sample study of SPN including 1798 cases reported a mortality of 15% and a decreasing annual rate of necrosectomies performed [13]. A 2007 study of the American College of Surgeons National Surgical Quality Improvement Program (ACS-NSQIP) database, a national registry for surgery and postoperative outcomes, indicated a lower 30-day mortality of 6.8% in 161 patients [14]. A later 2007–2012 ACS-NSQIP study of 1156 surgical necrosectomies indicated that mortality was 9.5%, with the modified Frailty Index (mFI) being a strong predictor of mortality and intensive care unit (ICU) complications [15]. National trends in incidence, mortality, and risk factors for ICU admission for patients undergoing operative necrosectomy have not been reported since description of the “step-up” approach.

We hypothesized that complications for surgically treated severe necrotizing pancreatitis patients decreased from baseline (2007–2010) during the period of adoption of the “step-up” approach (2011–2019). Our primary aim was to determine preoperative clinical factors for death and ICU Clavien-Dindo Class 4 (CD4) complications for surgically treated severe necrotizing pancreatitis patients in ACS-NSQIP during the “step-up” period. Our secondary aim was to determine time trends for the performance of SPN, preoperative risk factors, case rates, mortality, and CD4 complications for SPN during the “step-up” period.

## Methods

We performed a retrospective study using the ACS-NSQIP registry, which has 719 participating sites with 8,581,877 cases [16]. We identified patients undergoing operative pancreatic necrosectomy (Current Procedural Terminology (CPT) code 48,105) by surgeons from 2007 to 2019. This is the only CPT code for pancreatic necrosectomy and includes open necrosectomy as well as minimally invasive necrosectomies such as VARD. This was plotted as the number of cases, deaths and CD4 complications per year in ACS-NSQIP and as a percentage of annual NSQIP records. The preoperative variables collected included age, sex, modified 11-item frailty index (mFI), functional status, alcohol use (defined as 2 or more drinks in the previous 2 weeks), smoking history, history of congestive heart failure, history of myocardial infarction, steroid use, and emergency surgery status).

The mFI is a measure of frailty that has been predictive of operative outcomes in multiple studies and is calculated using the concept of “accumulating deficits” defined by variables available in the NSQIP database [17]. The 11 mFI variables are (1) nonindependent functional status; (2) history of diabetes mellitus; (3) history of chronic obstructive pulmonary disease or pneumonia; (4) history of congestive heart failure; (5) history of myocardial infarction; (6) history of percutaneous coronary intervention, stenting, or angina; (7) history of hypertension requiring medication; (8) history of peripheral vascular disease or ischemic rest pain; (9) history of transient ischemic attack or cerebrovascular event; (10) history of cerebrovascular accident with neurologic deficit; and (11) history of impaired sensorium. Functional status as assigned by the submitting center is defined by the patient’s independence in performing activities of daily living (ADLs) in the 30 days preceding surgery. Functional status 1 refers to an independent patient without the need for assistance to perform ADLs. Functional status 2 is partial dependence on assistance from another person. Functional status 3 is completely dependent on another person for ADLs. Nonindependent status indicates a functional status of 2 or 3.

The preoperative laboratory tests analyzed were white blood cell count (WBC), hematocrit (Hct), platelet count, serum sodium, blood urea nitrogen (BUN), creatinine, albumin, bilirubin, alkaline phosphatase, aspartate aminotransferase (AST), partial thromboplastin time (PTT), international normalized ratio (INR), and prothrombin time (PT).

Primary outcomes were postoperative complications stratified into Clavien-Dindo (CD) class 4 complications requiring ICU care including septic shock, myocardial infarction, cardiac arrest, pulmonary embolism, dialysis, reintubation, on ventilator for more than 48 h, and CD class 5 (death). Frailty, emergency surgery status, functional status and preoperative lab work (were evaluated by univariate and multivariate analyses as predictors of mortality. (NSQIP provides the last available result and duration of preoperative days, in this dataset laboratory results were obtained a mean of 2.4 ± 8.5 days preoperatively.) Post-SPN CPT codes for endoscopic necrosectomy (43,240, 48,999) and interventional radiology drainage procedures (10,030, 49,405, 49,406, and 49,407) were also collected.

Annual cases, deaths, and CD4 complications per year in ACS-NSQIP were reported both as absolute values and also as a percentage of annual NSQIP records. As the ACS-NSQIP registries adds new hospitals each year, including community hospitals as well as tertiary referral centers, a comparator group of pancreatoduodenectomies (Whipples) was identified (CPT codes 4815, 48,152, 48,153 and 48,154) to examine relative time trends in another pancreatic procedure. Whipples were selected as a comparator due to their likelihood of being performed at tertiary referral centers thought also likely to perform surgical necrosectomy.

Statistical analysis was performed using SPSS Version 28 (IBM, Armonk, NY). A *p* value less than 0.05 was considered statistically significant. Categorical variables were reported as a percentage of the total group and analyzed using Pearson’s chi-square and Fisher’s exact tests. Time trends of mFI, mortality and CD4 complications were performed using Pearson’s chi-square with the Mantel–Haenszel test. Nonparametric testing for significance of ordinal and continuous variables was confirmed with the Wilcoxon rank sum test. Listwise deletion was used if a case had missing data for any of the variables and that case was excluded from analysis. Logistic regression variables for mortality and complications were selected from prior literature, Kolbe et al.’s 2015 study of SPN in NSQIP [15]. ACS-NSQIP was used under the requirements of the UC San Diego Institutional Review Board and followed the ACS-NSQIP data use agreement.

## Results

**Fig. 1:**
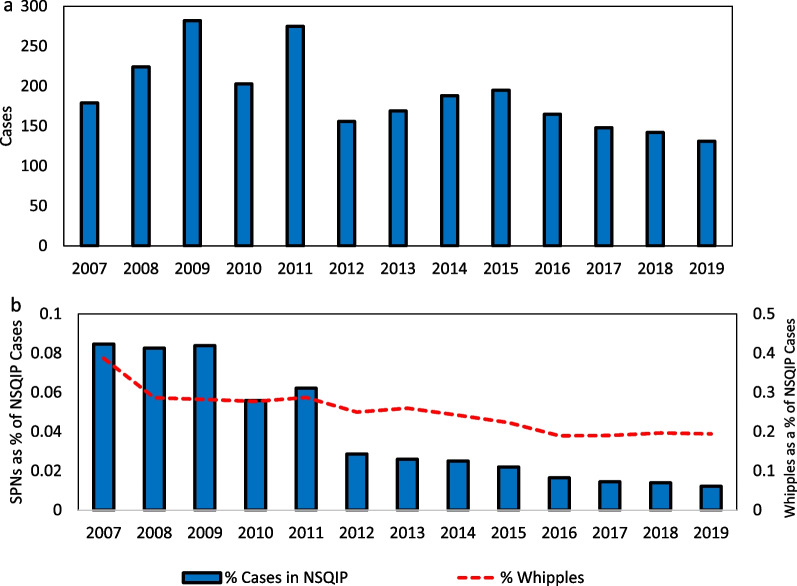
**a**: The number of cases of surgical necrosectomies in NSQIP from 2007 to 2019. **b**: Blue columns indicate the percentage of annual NSQIP records that were for surgical necrosectomies (SPNs) from 2007 to 2019. There was a 72% reduction in SPN from 2007–2010 to 2011–2019 (*p* < 0.001). The percentage of pancreatoduodenectomies (Whipples) of annual NSQIP records is plotted in red on the second axis, which decreased only 29% for the same period

A total of 2457 patients underwent SPN from 2007 to 2019. The trend of surgical necrosectomies decreased significantly from 0.09% (183 of 211,407) of all NSQIP cases in 2007–0.01% (135 of 1,076,441) in 2019 (*p* < 0.001, Fig. 1a). SPNs as a proportion of all NSQIP records annually are shown in Fig. 1b. There was a 72% reduction in ACS-NSQIP SPN cases from 2007–2010 to 2011–2019, while contemporaneous pancreatoduodenectomy procedures (“Whipples”) decreased 29% (*p* < 0.0001).

A total of 1722 (70.1%) SPN cases were male, the mean age was 54.2 ± 14.6 years, the mean BMI was 41.9 ± 12.3, 33.0% had diabetes, and 8.7% had alcohol use (Table 1). Increased age, non-white race, increased BMI, alcohol use, and functional status were univariate factors predictive of increased ICU-level complications. Non-white race, alcohol use, and functional status were univariate factors predictive of increased mortality.

**Table 1:** Demographics and comorbidities

	Clavien-Dindo 4 complication	Mortality	All cases
Number (%)	831 (33.8%)	212 (8.6%)	2457 (100%)
Mean age (± SD)	54.7 (± 14)	54.0 (± 14)	54.3 (± 14)
Mean BMI (± SD)	32.4 (± 10)	29.1 (± 8.1)	29.5 (± 8.6)
*Sex*			
Male	34.6%	5.8%	70.1%
Female	32.0%	2.8%	29.9%
Non-white race	33.1%	35.6%	27.8%
Alcohol use	14.4%	8.4%	8.7%
Smoker	23.1%	22.7%	22.7%
Diabetes	69.0%	32.0%	33.0%
Hypertension on meds	66.7%	62.6%	58.8%
CHF within 30 days	5.6%	5.8%	2.6%
MI within 30 days	0.5%	2.4%	0.8%
Chronic steroid use	3.7%	6.1%	4.4%
Emergency surgery	59.9%	49.5%	26.9%
*Functional status*			
Independent	52.2%	52.8%	73.8%
Partially dependent	8.9%	10.5%	10.3%
Totally dependent	38.8%	36.5%	15.1%

*CHF*, congestive heart failure, *MI*, myocardial infarction

**Fig. 2:**
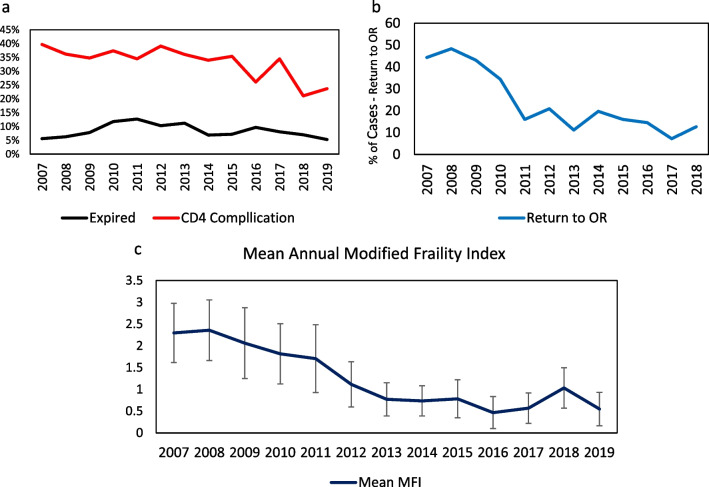
**a** There was an overall 65% in decrease in returns to the OR after SPN during the study period (*p* < 0.001). **b**. There was an overall 65% decrease in returns to the OR after SPN during the study period (*p* < 0.001). **c**. The annual mean modified frailty index (mFI) for SPN decreased significantly throughout the study period (*p* < 0.001). Error bar interval indicates one standard deviation

The CD4 complication rate decreased significantly throughout the study period (*p* < 0.001), while the mortality rate did not (*p* = 0.932, Fig. 2a. Overall, 33.8% of patients experienced a CD4 complication. Univariate analysis for CD4 complications and deaths indicated that nonindependent functional status (*p* < 0.001), modified frailty index (mFI) (*p* < 0.001) and emergency surgery status (*p* < 0.001) were highly significant. Preoperative BUN, serum albumin, alanine serum transferase (AST), relative leukocytosis, relative anemia, thrombocytopenia, and PTT (Table 2) were significant univariate predictors of mortality and ICU-level complications. The frequency of ICU-level complications in patients with CD4 complications is shown in Table 3. Failure to wean from the ventilator in less than 48 h was the most frequent complication at 63.7%.

**Table 2:** Laboratory values

	Clavien-Dindo 4 complication			Mortality		
	Yes	No	*p* value	Yes	No	*p* value
Sodium (mmol/l)	138.6 ± 5.6	136.8 ± 41	< 0.001*	137.0 ± 4.6	139.1 ± 17.0	< 0.001*
WBC × 10^9^ cells/l	16.4 ± 9.2	10.8 ± 6.0	< 0.001*	17.0 ± 10.1	12.3 ± 7.3	< 0.001*
BUN (mg/dl)	34.6 ± 23.5	15.1 ± 13.0	< 0.001*	39.0 ± 23.5	20.1 ± 183	< 0.001*
Creatinine (mg/dl)	1.7 ± 1.5	0.9 ± 0.8	< 0.001*	1.9 ± 1.5	1.1 ± 1.1	< 0.001*
Albumin (g/dl)	2.1 ± 0.7	2.8 ± 0.9	< 0.001*	2.0 ± 078	2.6 ± 0.9	< 0.001*
Bilirubin (mg/dl)	1.6 ± 2.1	0.9 ± 1.4	< 0.001*	2.5 ± 3.0	11 ± 1.5	< 0.001*
AST (U/l)	148 ± 129.8	35 ± 37.9	< 0.001*	173 ± 168	40 ± 60	< 0.001*
Alk phos (U/l)	153 ± 140	60 ± 95	0.972	173 ± 170	149 ± 133	0.125
Hct %	28.3 ± 5.2	31.6 ± 6.3	< 0.001*	28.3 ± 5.7	30.7 ± 6.1	< 0.001*
Platelets (k/μl)	284 ± 150	323 ± 145	< 0.001*	227 ± 136	318 ± 146	< 0.001*
PTT (sec)	35.4 ± 11.9	32.5 ± 8.6	< 0.001*	35.6 ± 11.6	33.4 ± 9.8	0.015
INR	1.4 ± 0.3	1.3 ± 0.2	< 0.001*	1.4 ± 11.6	1.3 ± 0.3	< 0.001*

*WBC*, white blood cell, *BUN*, blood urea nitrogen, *AST*, aspartate aminotransferase, *Alk phos*, alkaline phosphatase, *Hct*, hematocrit, *PTT*, partial thromboplastin time, *INR*, international normalized ratio

*p < 0.001, confirmed by Wilcoxon rank sum test

**Table 3:** Rates of Clavien-Dindo class 4 complications for necrosectomy patients

Clavien-Dindo class 4 complication	Frequency (%)
Failure to wean from the ventilator < 48 h	63.7
Septic shock	14.4
Reintubation	7.2
Renal failure	3.1
Pulmonary embolism	1.8
Cardiac arrest	2.2
Myocardial infarction	0.6

The number of cases requiring a return to the operating room after the index surgical procedure is shown in Fig. 2b. There was a 65% reduction cases requiring a return to the operating room when comparing 2007–2010 to 2011–2019 (*p* < 0.001). The mean mFI decreased annually from 2.3 in 2007 to 0.6 in 2019 (*p* < 0.0001) (Fig. 2c).

**Fig. 3:**
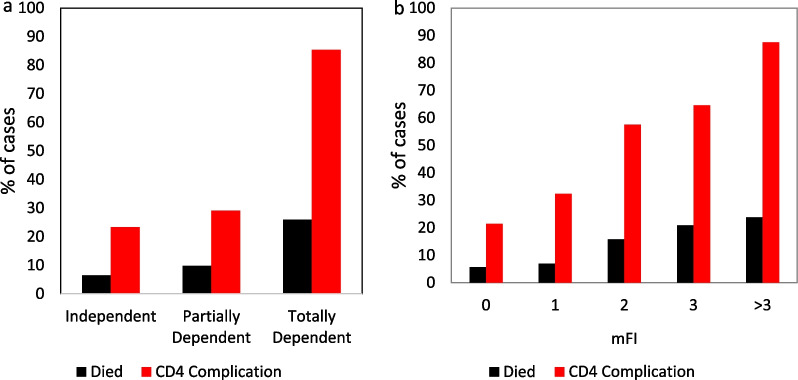
**a**: Rates of Clavien-Dindo Class 4 complications and mortality for SPN categorized by peak functional status. **b**: The modified frailty index (mFI) by mortality (*p* < 0.001) and Clavien-Dindo class 4 complications (*p* < 0.001) for SPN

Preoperative functional status had a significant association with CD4 complications (*p* < 0.001) and mortality (*p* < 0.001, Fig. 3a). The modified frailty index (mFI) was also a significant predictor of increased mortality (*p* < 0.001) and CD4 complications (*p* < 0.001, Fig. 3b).

Predictors of CD4 complications by binary logistic regression are shown in Table 4. These were mFI (*p* ≤ 0.0001), low serum albumin (*p* < 0.0001), BUN (*p* < 0.0001), elevated AST (*p* = 0.0002), and emergency surgery status (*p* < 0.0001). Predictors of mortality by binary logistic regression were mFI (*p* = 0.040), BUN (*p* < 0.001), serum albumin (*p* < 0.001), elevated AST (*p* = 0.003), and emergency surgery status (*p* < 0.0001).

**Table 4:** Logistic regression analysis

	Odds ratio (95% C.I.)	*p* value
*Clavien-Dindo 4 complication*		
Modified frailty index	1.58 (1.37–1.68)	< 0.0001
Emergency case	2.78 (2.20–3.50)	< 0.0001
Low serum albumin	2.68 (2.46–2.89)	< 0.0001
Elevated BUN	1.04 (1.03–1.05)	< 0.0001
Elevated AST	1.004 (1.002–1.006)	0.0002
*Mortality*		
Modified frailty index	1.21 (1.12–1.36)	0.040
Emergency case	3.20 (2.32–4.30)	< 0.0001
Low serum albumin	2.96 (2.57–3.40)	< 0.0001
Elevated BUN	1.02 (1.02–1.03)	< 0.0001
Elevated AST	1 (1.00–1.01)	0.0003

## Discussion

The role of SPN in severe necrotizing pancreatitis lacks broadly accepted guidelines or clear indications for the various nonoperative and operative procedures beyond an initial nonoperative, “step-up” approach [11]. This study is the largest retrospective collection of SPN cases, using a cross-sectional database of patients in the US, Canada, the UK, Europe and Australia, with 2457 cases recorded between 2007 and 2019, in which we examined preoperative predictors of CD4 complications and mortality. We found a low and stable mortality rate for SPN, with a decreasing rate of ICU complications. The frequency of SPN in the ACS-NSQIP decreased significantly after 2010, while the patients undergoing SPN had decreasing frailty scores throughout the study period. There may be a selection bias that SPNs may be occurring mostly at academic, tertiary referral centers, which may have been faster to join ACS-NSQIP than later-joining centers; however, the rate of decrease in performance of SPNs was significantly more rapid after 2010 than the decrease in pancreaticoduodenectomies during the study period, indicating that fewer SPNs are being performed in ACS-NSQIP contributor hospitals.

ACS-NSQIP does not allow tracking of preoperative procedures that may have been performed before the SPN, so compliance with the “step-up” approach cannot be exactly determined. After SPN, additional endoscopic or interventional radiology procedures for WOPN are uncommon. CPT codes for endoscopic procedures post-SPN were quite rare, possibly indicating that SPN was a definitive management. Unfortunately, there is no available CPT code for endoscopic pancreatic necrosectomy. The 2010 PANTER trial randomized patients into either primary SPN or a step-up MIS approach to a possible SPN using percutaneous and/or endoscopic transgastric drainage, followed by a second drainage procedure if the initial procedure failed after 72 h, and then VARD if there was still no improvement after a further 72 h. In the step-up group, 26 of 43 (60%) underwent SPN after percutaneous drainage, 24 received VARD and 2 had open necrosectomies. Further procedures were required in 33% of step-up cases. In the initial necrosectomy group, 42% required additional laparotomies for additional necrosectomies, and 33% required additional percutaneous drainage procedures. While ACS-NSQIP cannot determine exactly who underwent a “step-up” approach, our study indicates that (1): the number and proportion of cases of SPNs decreased significantly after 2010; (2): the number of SPN patients requiring a return trip to the operating room also decreased. We believe this is evidence of the adoption of an MIS approach such as that used in the PANTER algorithm. The decrease in complications after 2010 may be further evidence for a greater use of MIS approaches such as VARD versus open necrosectomy but may also represent a selection bias of managing frail patients via endoscopic and interventional radiologic procedures without SPN.

There was a significant decrease in frailty index scores after 2010, indicating that less frail patients with a lower risk of complications were being selected for SPN, which may also have contributed to fewer complications. The modified Frailty Index became less predictive of CD4 complications and mortality with lowered odds ratios compared to an earlier ACS-NSQIP study for the period of 2007–2012 [15]. This may also be further evidence that less frail patients were being selected for SPN. Whether more frail patients were managed with interventional radiology placed drains or endoscopic debridement without SPN cannot be determined by this paper. We believe the PANTER trial may have influenced providers not only to consider nonoperative procedures before surgical procedures but also to avoid SPN in frail patients altogether.

The PANTER trial showed that the step-up approach did reduce complications but did not significantly affect mortality. Similarly, our study showed that while complications did decrease during the study period, the death rate remained stable. This may indicate that the mortality of severe infected pancreatic necrosis may not be greatly affected by current SPN procedures due to death being caused by existing or non-modifiable factors such as multisystem organ failure, late presentation or medical comorbidities. Frailty and nonindependent functional status were important predictors of mortality and should be significant adverse risk factors for surgeons considering offering SPN after failure of nonoperative management of severe necrotizing pancreatitis. ACS-NSQIP rules indicate the emergency surgery data element should be assigned as a “YES” if the surgeon and/or anesthesiologist reported the case as emergent, “usually performed within a short interval of time between patient diagnosis or the onset of related preoperative symptomatology,” and “the patient’s well-being and outcome is potentially threatened by unnecessary delay and the patient’s status could deteriorate unpredictably or rapidly.” The increased mortality and complications for emergency performance of SPN should also be considered an adverse risk factor by surgeons. If nonoperative alternatives to urgent SPN are an option, these may in fact be safer. However, we cannot tell from ACS-NSQIP whether suspicion of complications of severe acute pancreatitis, such as perforated viscus, hemorrhage, bowel ischemia or uncontrolled sepsis, precipitated a decision to perform urgent SPN [18].

There are several limitations to our study. We cannot tell the exact SPN procedure that was performed or the order of procedures, including whether the step-up approach was used for any particular patient. We believe the decrease in SPNs is indirect evidence for the acceptance of the PANTER trial results after 2010 and not due to other possible issues with the ACS NSQIP database in capturing CPTs for SPN; however, there may have been other trends in adopting more MIS and nonoperative approaches not related to the PANTER trial. We do not have information on the timing of the procedures in relation to the time of onset of severe necrotizing pancreatitis. We also do not know if there was evidence of infected necrotizing pancreatitis or what the abdominal anatomy of the infected collections might have been. CT scan results are not available within the database. A number of adverse outcomes were not included in the ACS-NSQIP, such as pancreatic fistulae, delayed abdominal closure, incisional hernias, new onset diabetes, need for enteral and parenteral nutrition, need for enzymatic supplementation and prolonged ICU stay. Although we saw a decrease in necrosectomies, the ACS-NSQIP data are submitted only from hospitals and countries that are participating in the ACS-NSQIP and may not represent a statistically valid, nationally representative sample, although the sample is quite large.

This updated analysis of ACS-NSQIP since the study by Kolbe et al. for the 2007–2012 period does indicate that there has been a significant decrease in the performance of SPNs [15]. SPN may not be obsolete, as there is the ability to select patients for a step-up approach based on their frailty, functional status, and laboratory results to achieve reduced complications. Given the relative rarity of SPN procedures, we recommend that SPNs be included in prospectively collected registries, such as current and planned EGS registries, including imaging, anatomic and procedural variables, to obtain more predictive outcome data.

## Conclusion

The number of surgeon-performed SPNs in the US has significantly decreased since 2010. Complications and returns to the OR for repeated procedures have been reduced, and less frail patients are being selected for surgery. These trends are likely due to increased adoption of the “step up” approach after 2010. The 30-day postoperative mortality rates remained stable despite decreased preoperative frailty. Further prospective studies to determine indications for routine and urgent SPN and the optimal sequence of interventional radiologic, endoscopic and minimally invasive surgical operative debridement techniques as part of the “step-up” paradigm are warranted.

## Supplementary Information

**Additional file 1:** **Fig. S1**. Representative CT scan images of a patient with infected, walled-off pancreatic necrosis (WOPN). **Fig. S2**. Planned incision for VARD, laparoscopic VARD view of pancreatic necrosum. Wound appearance post-VARD before wound closure. **Fig. S3**. Pancreatic necrosum extracted via video-assisted retroperitoneal debridement (VARD), patient from Fig. 1.

## Data Availability

The data that support the findings of this study are available from the American College of Surgeons (ACS) National Surgery Quality Improvement Program (NSQIP) database, but restrictions apply to the availability of these data, which were used under a User Agreement for the current study and thus are not publicly available. Data are, however, available from the authors upon reasonable request and with permission of ACS-NSQIP.

## Abbreviations

ACS: American college of surgeons
AST: Aspartate aminotransferase
BUN: Blood urea nitrogen
CD4: Clavien-Dindo class 4 complication
CHF: Congestive heart failure
CPT: Current procedural terminology code
EGS: Emergency general surgery
Hct: Hematocrit
ICU: Intensive care unit
INR: International normalization ratio
mFI: Modified frailty index
MI: Myocardial infarction
MIS: Minimally invasive surgery
MOF: Multiple organ failure
NSQIP: National surgery quality improvement program database
PANTER: Acute necrotizing pancreatitis trial
PTT: Partial thromboplastin time
SPN: Surgical pancreatic necrosectomy
VARD: Video-assisted retroperitoneal debridement
WBC: White blood cell count
WOPN: Walled-off pancreatic necrosis
