# Supplementary material for: Decreasing use of pancreatic necrosectomy and NSQIP predictors of complications and mortality
Source: World J Emerg Surg. 2022 Dec 12;17:60. doi: 10.1186/s13017-022-00462-8 (PMC9743619; doi:10.1186/s13017-022-00462-8)
Supplement: Supplementary file 1 — Additional file 1: Fig. S1. Representative CT scan images of a patient with infected, walled-off pancreatic necrosis (WOPN). Fig. S2. Planned incision for VARD, laparoscopic VARD view of pancreatic necrosum. Wound appearance post-VARD before wound closure. Fig. S3. Pancreatic necrosum extracted via video-assisted retroperitoneal debridement (VARD), patient from Fig. 1. [file 13017_2022_462_MOESM1_ESM.docx]

**SUPPLEMENTARY MATERIAL**

**Figure S1.**


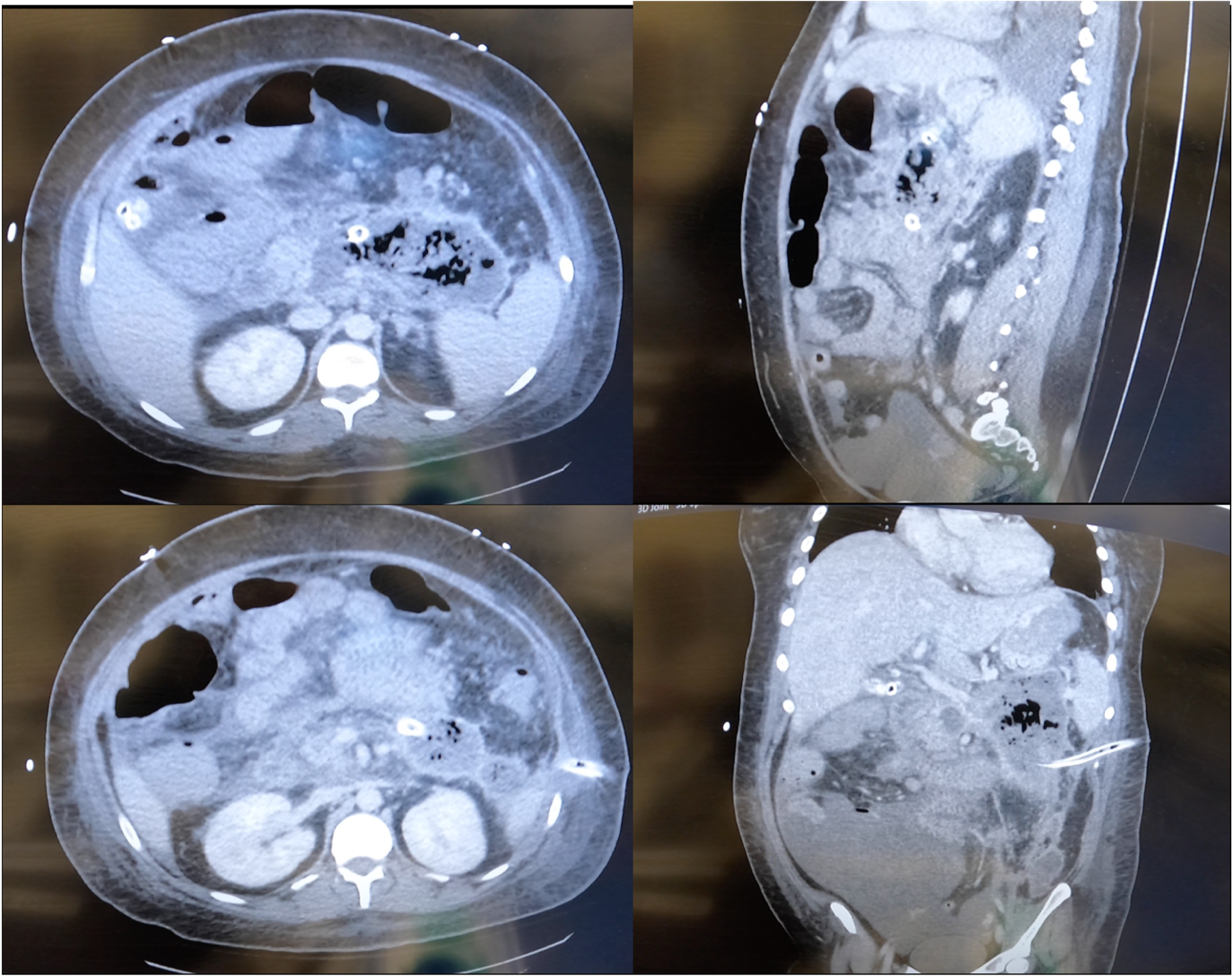


**Figure S1 – Representative CT scan images of a patient with infected, walled-off pancreatic necrosis (WOPN).**

**Figure S2.**


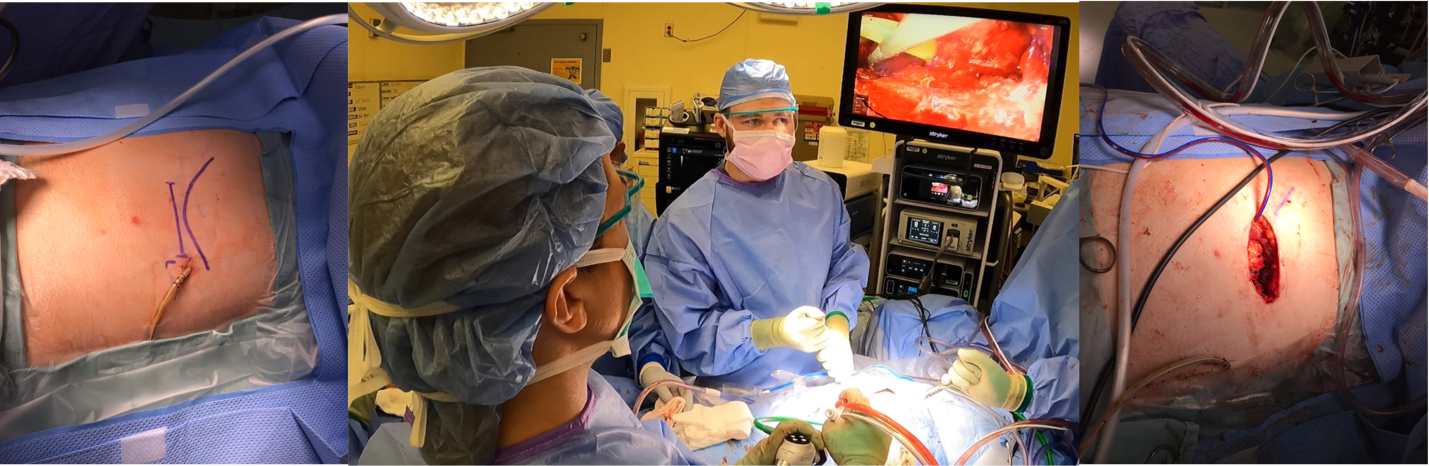


**Figure S2. Planned incision for VARD, laparoscopic VARD view of pancreatic necrosum. Wound appearance post-VARD before wound closure.**

**Figure S3.**


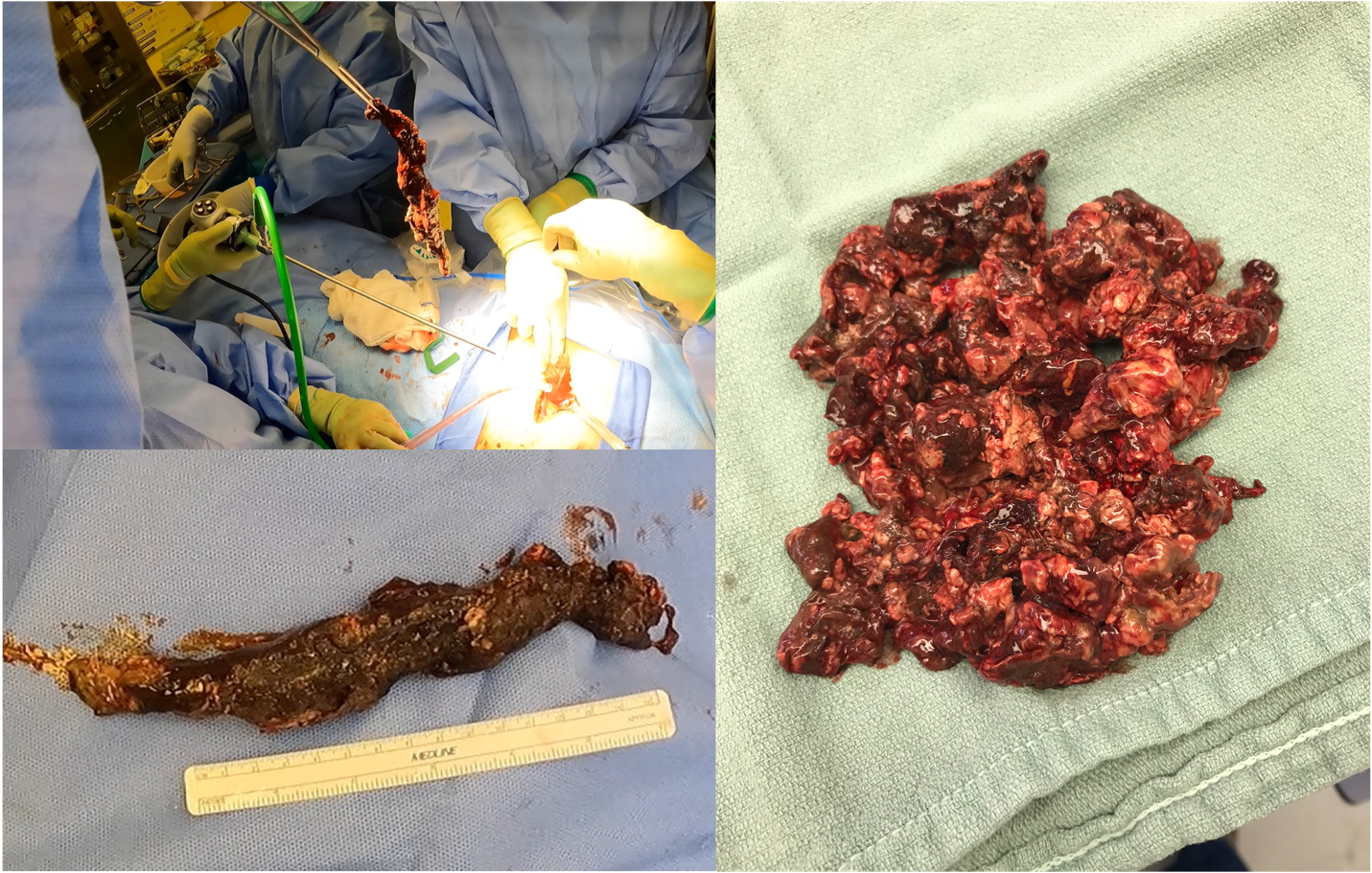


**Figure S3. Pancreatic necrosum extracted via video-assisted retroperitoneal debridement (VARD), patient from Figure 1.**
